# Efficient Dataset Creation for MEMS-Based Magnetic Sensor Systems in Intelligent Transportation Applications

**DOI:** 10.3390/s25247407

**Published:** 2025-12-05

**Authors:** Michal Hodoň, Peter Šarafín, Lukáš Formanek, Andrea Kociánová

**Affiliations:** 1 Department of Technical Cybernetics, Faculty of management Science and Informatics, University of Žilina, Univerzitna 8215/1, 010 26 Žilina, Slovakia; peter.sarafin@fri.uniza.sk (P.Š.); lukas.formanek@fri.uniza.sk (L.F.); 2 Department of Highway and Environmental Engineering, Faculty of Civil Engineering, University of Žilina, Univerzitna 8215/1, 010 26 Žilina, Slovakia; andrea.kocianova@uniza.sk

**Keywords:** magnetic sensors, vehicle detection, data annotation, data fusion, intelligent transportation systems

## Abstract

This article describes the innovative use of an advanced annotation tool designed specifically for creating datasets tailored to MEMS (Micro-Electro-Mechanical Systems) sensor systems for the intelligent transportation domain. By optimizing the data annotation process, this tool significantly enhances the efficiency and accuracy of dataset development, which is critical for the optimal performance and reliability of MEMS-based applications. The tool was tested with a specialized sensor system based on magnetometers for traffic flow monitoring, demonstrating its practical applications and effectiveness in real-world scenarios. The proposed approach offered a clear improvement over manual labelling by reducing the time needed per event and increasing the number of events that could be processed, without compromising the consistency of the assigned labels. The discussion includes a detailed overview of the tool’s features, its integration into existing workflows, as well as the benefits it offers engineers and researchers in the field of sensor technology.

## 1. Introduction

In the development of intelligent systems based on MEMS (Micro-Electro-Mechanical Systems) sensors, the creation of high-quality annotated datasets plays a crucial role in achieving reliable performance and accurate signal interpretation. However, the process of labelling sensor data—particularly in multimodal environments—can be complex and time-consuming. To address this challenge, we have developed a dedicated software tool designed specifically for annotating data from MEMS-based sensor systems, with a focus on traffic flow monitoring applications. The tool supports synchronized playback and annotation of data streams from multiple sources, including magnetometers, radar (Sierzega SR4), and outputs from vehicle detection algorithms. Implemented in Java for the Windows platform, the application integrates JavaFX for the user interface, VLCJ for video playback, and ChartFX for signal visualization. Annotators can interact with a synchronized camera feed and assign detected objects to specific vehicle classes; each linked to corresponding magnetic signal records. This enables the generation of well-structured, labelled datasets suitable for training and validating detection or classification algorithms and demonstrates the tool’s potential for improving annotation efficiency in real-world sensor-based applications.

In modern MEMS-based sensor system development, creating high-quality annotated datasets remains a central challenge due to the need to align signals from multiple modalities—inertial sensors, magnetometers, radar, and video—and label them accurately for supervised model training. Recent systems have begun integrating software tools that support smart, synchronized annotation of combined sensor streams to improve dataset integrity [1]. Semi-supervised annotation of synchronized inertial and video sensor data has been shown to significantly reduce labelling effort while improving temporal consistency and dataset reliability [2]. Robust annotated datasets remain foundational for developing MEMS-based sensor systems, particularly in mobility and transportation applications. Transportation mode detection using multimodal smartphone sensor data demonstrates that the fusion of inertial and contextual measurements substantially improves classification accuracy when supported by recurrent neural networks [3]. Further advances using deep residual and recurrent network architectures confirm that accurate temporal alignment and structured feature fusion across multiple sensor streams are essential for robust and scalable transportation pattern recognition [4]. Beyond algorithmic performance, the importance of structured data handling, transparency, and trust in IoT-enabled sensing environments has been emphasized. Frameworks addressing data observability and accountability demonstrate that clearly defined data pipelines and annotation workflows are essential when managing heterogeneous sensor data at scale [5]. The importance of synchronized multi-sensory datasets is further demonstrated through large-scale vehicular and mobility datasets. Transportation datasets built from multimodal smartphone sensors illustrate how high-quality sensor alignment supports generalizable transportation mode recognition across diverse usage scenarios [6]. Authors in [7] review IoT-connected traffic sensor systems and highlight how accurate sensor data annotation supports edge intelligence, object tracking, and real-time deployment in smart-city infrastructures. Synchronized annotation tools are identified as essential enablers of such deployments. In vehicular cyber-physical systems, time-synchronized datasets combining video, inertial, GPS, and magnetic sensors enable reliable benchmarking of sensor-fusion and perception algorithms under real-world traffic conditions [8]. Despite the availability of synchronized datasets, annotation remains a major practical bottleneck. Smart annotation tools that integrate synchronized sensor streams with human-in-the-loop support have been shown to significantly reduce manual labelling effort while improving temporal consistency and label quality [9]. Complementary intelligent dataset construction pipelines further demonstrate that tool-assisted approaches improve noise filtering, scalability, and downstream machine-learning performance [10]. Advanced vehicle detection methods based on cross-scale feature fusion further demonstrate that balanced and accurately annotated datasets across different object scales are essential for achieving consistent detection accuracy in complex traffic scenes [11]. Large-scale annotation pipelines, such as those combining LiDAR tracking and human-in-the-loop correction, achieved three- to four-fold speed improvements over manual annotation, demonstrating scalability benefits [11]. While not MEMS-specific, these scalable workflows inform our tool’s semi-automated architecture. Similarly, multimodal annotation tools for ADAS datasets (e.g., ezLabel) support synchronized video and object labelling based on detection outputs, reducing overall manual annotation time [11]. Dataset quality has been repeatedly identified as a dominant factor influencing perception system performance. Comprehensive reviews of vehicle detection under adverse environmental conditions highlight that annotation accuracy, dataset diversity, and sensing quality critically affect detection robustness in real-world deployments [12].

Beyond conventional visual sensing, alternative dataset modalities also play an increasingly important role. Large-scale acoustic datasets for emergency vehicle and traffic sound detection demonstrate that carefully labeled sensor data enable highly accurate detection systems for intelligent transportation and public safety applications [13]. Event-based and hybrid vision datasets providing synchronized event streams and frame-based imagery with precise ground-truth annotations further illustrate the growing demand for high-quality, temporally aligned multimodal datasets in automotive detection and tracking tasks [14]. Our dataset tool similarly supports metadata-driven labelling to facilitate algorithmic vehicle-type classification. Taken together, these works illustrate the demand for specialized annotation tools tailored to MEMS-based sensor systems in traffic environments. The following sections describe the system architecture, implementation details, and practical validation through real-world use cases.

Recent work in Intelligent Transportation Systems shows a rapidly increasing demand for reliable and well-annotated datasets that support autonomous driving, traffic modeling, and intelligent infrastructure. Authors in [15] demonstrated that high-speed autonomous vehicles can significantly improve their cruising performance when supported by an integrated deep reinforcement learning framework. Their study highlights that such learning-based systems depend strongly on high-quality training data to handle fast-changing traffic situations safely and efficiently. Authors in [16] analyzed large-scale vehicle trajectory data to investigate differences in lane-changing behaviour on highways. Their results show that detailed, precisely labelled movement data are essential for understanding how drivers behave in real traffic and for developing more realistic traffic behaviour models. Authors in [17] examined how cyber-attacks and external disturbances can influence traffic stability by using a multi-phase hydrodynamic traffic model with a self-stabilizing control protocol. Their findings emphasize the importance of accurate traffic data for designing robust control strategies that can maintain stability even under abnormal or hostile conditions. In [18], the authors developed a supercapacitor-based power unit for event-driven wireless sensor nodes, showing how low-power sensing platforms can support long-term traffic monitoring in distributed environments. Together, these studies show that modern ITS applications rely heavily on accurate, multimodal, and well-annotated datasets, which motivates the development of the specialized annotation tool presented in this work. Recent studies demonstrate the growing importance of magnetic and multisensory data in intelligent transportation and smart-parking applications. Magnetometer-based vehicle detection systems have been shown to reliably identify vehicles in individual parking spaces, highlighting the need for accurate magnetic data processing and placement constraints [19]. Multisensory fusion approaches for situational awareness in V2V communication combine imaging, V2V data, GPS, and magnetometer signals to improve vehicle identification accuracy [20]. Road-embedded magnetometers have also been used for vehicle localization and lane-level tracking, demonstrating that magnetic signatures contain sufficient spatial information for estimating vehicle position [21]. In smart-parking systems, combining magnetic sensors with UWB channel features improves detection performance in weak magnetic regions and reduces false decisions [22]. Magnetic sensor arrays further enable the extraction of unique magnetic signatures for vehicle reidentification and lane position estimation [23], while sensor-fusion methods using accelerometers and magnetometers support detailed vehicle classification and axle-based feature extraction [24]. This study presents several contributions. First, it presents a multimodal annotation tool specifically optimized for MEMS-based magnetic sensor systems, capable of integrating magnetic, radar, and video data streams within a synchronized graphical interface. The system implements timestamp-based synchronization and adaptive filtering, ensuring millisecond-level temporal alignment across heterogeneous data sources. The inclusion of adaptive filtering, peak-based event segmentation, and automated event pre-detection provides a semi-automatic workflow that substantially reduces operator workload compared to manual magnetic signal labelling. A large-scale labelled dataset was created, comprising more than 50,000 vehicle records collected from 36 h of real-world measurements and categorized into five distinct vehicle classes. The proposed workflow was further evaluated in terms of annotation efficiency and reproducibility, demonstrating over a twofold reduction in labelling time compared with manual processing while maintaining accuracy above 95%. The tool serves as an open and extensible framework for future AI-assisted annotation, enabling intelligent, semi-automatic dataset generation for smart transportation applications. These contributions highlight the originality of the proposed approach beyond existing annotation frameworks such as EasyLabel [7] or ezLabel [11], extending their concepts toward resource-constrained MEMS-based traffic monitoring nodes.

## 2. Data Measurement

This work builds directly on our previously published research, where we introduced a traffic monitoring system based on magnetometer sensing and evaluated its performance under various installation and traffic conditions [25]. While the system demonstrated high detection accuracy and robustness, the process of data labelling and result validation remained a manual and time-consuming task. The need for structured, high-quality datasets became increasingly apparent—particularly to support algorithm development, long-term testing, and model transferability. To address this gap, the present study introduces a custom annotation tool that enables synchronized handling of sensor signals, video footage, and detection algorithm outputs. By enhancing the data annotation workflow, the tool significantly improves the usability of the magnetometer-based system and supports the creation of reliable training and evaluation datasets for further development in the field. Among the tested magnetometers in our previous work [25], the PNI RM3100 (PNI Sensor Corporation, San Jose, CA, USA) demonstrated the highest detection performance, particularly in terms of signal clarity, sensitivity, and noise robustness. Compared to lower-cost sensors like the HMC5883L (Honeywell, Phoenix, AZ, USA) or LIS3MDL (STMicroelectronics, Plan-les-Ouates, Geneva, Switzerland), the RM3100 provided significantly cleaner magnetic signatures across all axes, which improved the reliability of vehicle detection and classification. Its high dynamic range and low noise characteristics make it especially well-suited for real-time traffic monitoring applications, even in the presence of environmental noise and varying vehicle sizes.

To capture the magnetic signatures of passing vehicles, the PNI RM3100 digital magnetometer was installed in a roadside configuration. The sensor was mounted at ground level near the lane centreline to maximize sensitivity to ferromagnetic vehicle components. The system was configured with a sampling frequency of 250 Hz, providing high-resolution time-series data for the X, Y, and Z magnetic field axes. Data acquisition was performed in real traffic conditions, capturing a range of vehicle types under varying environmental influences.

The raw magnetic field data, denoted as $x\left[n\right]$, often contain low-frequency drift and high-frequency noise, which can obscure vehicle-induced events. Therefore, the recorded signals were preprocessed using a two-stage filtering pipeline. The raw magnetic field signal for each axis, denoted as $x\left[n\right]$, was first processed using a high-pass filter to remove the DC component and suppress long-term baseline variations:

(1) $$x_{HP}\left[n\right]=x\left[n\right]-\frac{1}{N}\sum_{k=n-N+1}^{n}x\left[k\right]$$

This step effectively eliminates the baseline offset in the measured signal. Next, a low-pass filter was applied to reduce high-frequency noise and smooth the signal:

(2) $$x_{LP}\left[n\right]=\alpha x_{HP}\left[n\right]+\left(1-\alpha \right)x_{LP}\left[n-1\right],\;\;0<\alpha <1$$

This exponential moving average produces a cleaner signal suitable for subsequent analysis. To enhance the detection of transient events (magnetic disturbances caused by vehicle passages), the signal energy over a sliding window of M samples was computed as

(3) $$E_{n}\left[n\right]=\sum_{k=n}^{n+M-1}x_{LP}^{2}\left[k\right]$$

**Figure 1:**
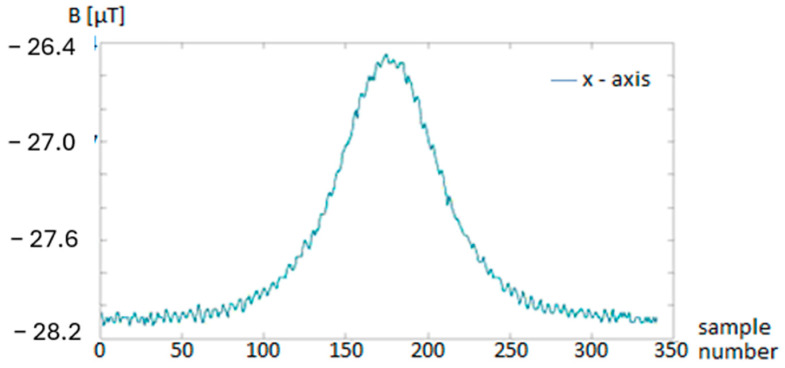
Raw measured data—magnetic flux density.

**Figure 2:**
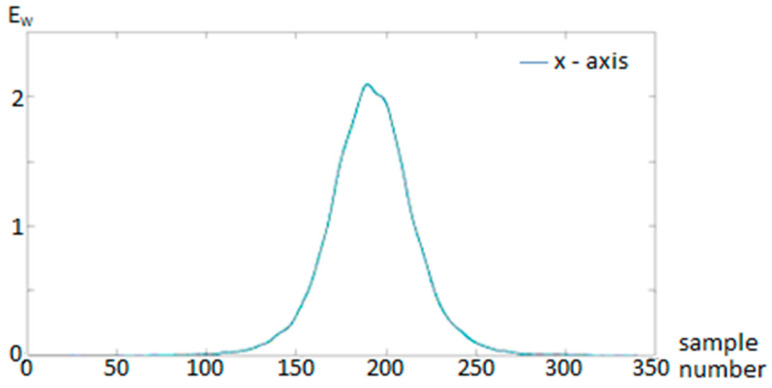
Filtered data—windowed energy E_W_.

This derived energy signal $E_{n}\left[n\right]$ served as the primary feature for vehicle detection (Figure 1 and Figure 2). It serves as a robust feature that highlights the presence of magnetic disturbances caused by passing vehicles.

Vehicle detection was performed by identifying peaks in the energy signal that correspond to significant magnetic disturbances. A peak was considered a valid detection if it satisfied the following criteria:

- Minimum peak height: $E_{n}\left[n\right]>m$;
- Minimum distance between peaks: ∆n≥d;
- Minimum signal duration above threshold: $T_{signal}>t$.

**Figure 3:**
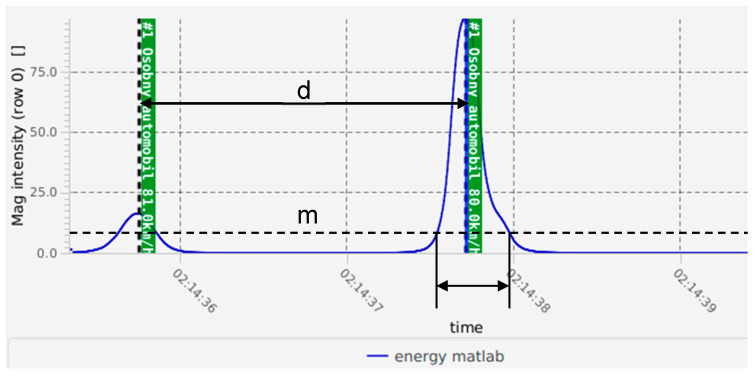
Vehicle detection rules.

The first condition ensures that only significant peaks, associated with actual vehicles, are considered. The second condition avoids multiple detections from the same vehicle by enforcing a minimum distance d (in samples) between adjacent peaks. The third condition ensures the signal remains above the detection threshold m for at least t milliseconds, filtering out short-lived noise spikes. An example of data record can be seen in the Figure 3.

To further stabilize the feature extraction process, a windowing function of length of N=512 samples was applied around each detected peak. This window isolates the vehicle event and prepares the segment for downstream classification or labelling.

Formally, a vehicle event is registered if

(4) ∃n0:En0<m, and En>θ ∀n∈n0−δ, n0+δ, δ=t2

In this context:

- N defines the total number of samples in the analysis window, centred around the detected peak.
- $n_{0}$ is the index of the detected local maximum in range.
- δ is half the window size, i.e., δ=N2, defining the extent of samples before and after the peak.
- θ is a reference threshold used to ensure the signal remains consistently above background noise.

**Figure 4:**
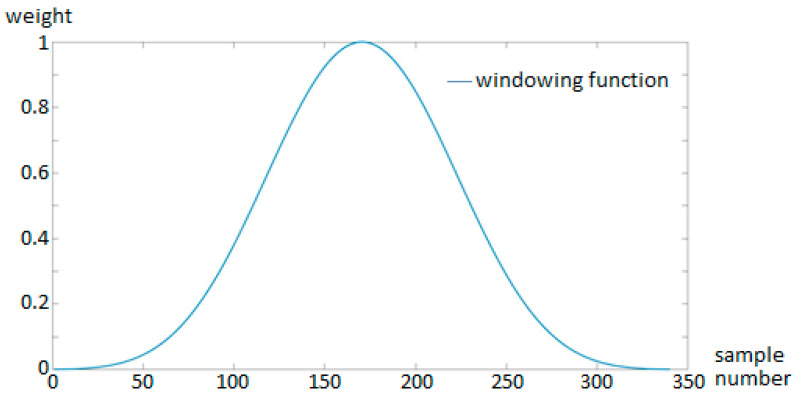
Filtered signal after the application of windowing function.

This framework enabled the system to reliably detect individual vehicle passages and assign them for further annotation using synchronized video and detection algorithm outputs. The filtered signal after the application of windowing function can be seen in Figure 4. The applied windowing function, illustrated in Figure 4, emphasizes the information at the centre of each time window while gradually suppressing contributions toward the edges. Since the window has a fixed length, the measured signal within it inherently depends on the properties of the passing object, such as its length and speed. The application of such a window is crucial, as it ensures that the analysis primarily focuses on the data near the window centre

Pseudocode of magnetic signal processing and vehicle detection can be seen in Algorithm 1.
**Algorithm 1.** Pseudocode of magnetic signal processing and vehicle detection algorithmInput: x[n]     - raw magnetic signal (from one axis) N_HP     - window size for high-pass filter α      - smoothing factor for low-pass filter M_energy     - window size for energy computation m      - minimum peak height d      - minimum peak distance (samples) t      - minimum duration above threshold (ms or samples)  θ       - reference signal threshold N_window   - number of samples for windowing (centered around peak) Output: DetectedVehicles—list of detected vehicle segments Step 1: High-pass filtering For each sample n:  x_HP[n] = x[n]—average(x[n − N_HP + 1 to n]) //High-pass filtered signal Step 2: Low-pass filtering (exponential moving average) x_LP[0] = x_HP[0] For each sample n > 0:  x_LP[n] = α * x_HP[n] + (1 − α) * x_LP[n − 1]  //Low-pass filtered signal Step 3: Compute signal energy For each sample n:  E[n] = sum over k = n to n + M_energy − 1 of x_LP[k]^2 //Energy of the signal Step 4: Find local maxima Initialize DetectedVehicles = [] For each n where E[n] is a local maximum:  //m—minimum height of energy peak  If E[n] > m AND time since last peak ≥ d:  //d—required minimum of samples   If E[n − δ to n + δ] > θ for duration ≥ t:    // θ—energy treshold    segment = x_LP[n − N_window/2 to n + N_window/2]    Add segment to DetectedVehicles Return DetectedVehicles

Once the signal filtering and vehicle detection procedures were implemented and validated, the resulting detection events served as input for the next phase of the system pipeline—data labelling and integration. To enable further analysis and supervised learning, each detected vehicle event needed to be associated with additional contextual information, including video footage and radar measurements. This step was essential for enriching the dataset with reliable ground truth and for supporting classification tasks based on magnetic signal patterns. The following section describes the tools and procedures developed for synchronizing, annotating, and managing these multimodal data sources.

## 3. Data Labelling and Integration

All data processing, detection, and labelling steps were integrated into a custom software tool developed for research purposes. The software was implemented in Java 13.01, using JavaFX 13.02 for the graphical user interface, VLCJ 4.7 for synchronized video playback, and ChartFX 8 for real-time signal visualization. The architecture supported loading of multi-channel time-series data, applying preprocessing filters, performing event detection based on defined parameters, and managing synchronized video streams. Annotators could browse through the timeline, adjust detection thresholds, and label each segment interactively. Exported datasets contained time-aligned sensor data, detection metadata, and video references, structured for downstream use in machine learning workflows and algorithm benchmarking.

**Figure 5:**
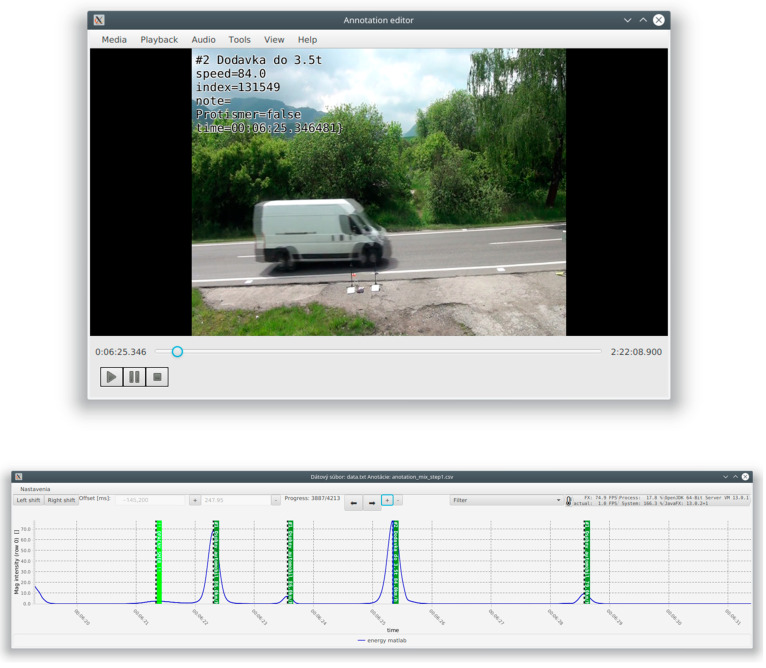
GUI of the developed annotation tool.

**Figure 6:**
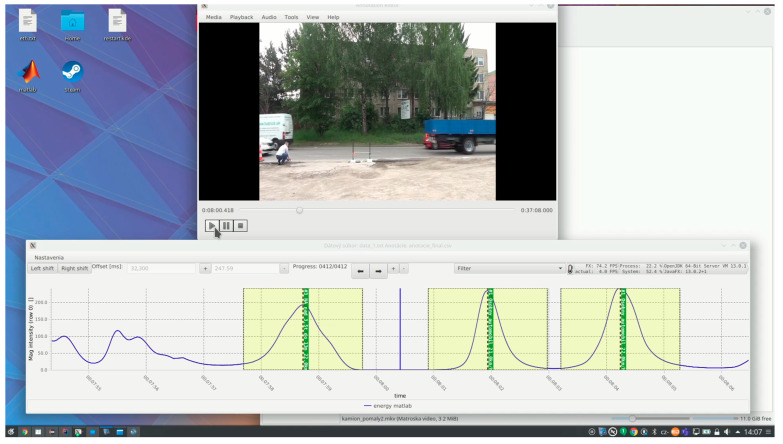
Application of the detection algorithm.

Following detection, each segmented event was synchronized with corresponding video recordings and radar data (Sierzega SR4) using timestamps. Annotators were able to view the synchronized camera footage and assign each detected event to a specific vehicle category (e.g., car, van, truck, bus). The annotation tool allowed the association of each labelled vehicle with its corresponding magnetic signature, creating a structured dataset for further analysis and machine learning. Graphical user interface of the developed annotation tool is shown in the Figure 5 and the application of the detection algorithm is shown in the Figure 6.

The system included detailed and well-defined implementation mechanisms. The magnetometer, radar, and video streams were synchronized using a common timestamping framework derived from a shared, millisecond-precision real-time clock. Magnetic and radar measurements were stored in CSV files, while video recordings were encoded in MP4 format and paired with JSON metadata specifying event indices, class labels, and alignment parameters. The software employed a stream-oriented, incremental loading architecture in which only the active segment of the dataset was held in memory, with the remaining data accessed through buffered disk reads. This approach ensured stable performance when working with multi-gigabyte datasets and prevented memory saturation during interactive navigation. Together, these mechanisms provided a transparent and fully reproducible workflow for multimodal data synchronization, annotation, and export.

To complement the reported dataset size, several indicators were introduced to quantify the annotation efficiency and labelling accuracy of the proposed system. A controlled evaluation with three independent annotators was conducted using a stratified subset of 200 vehicle events. The average annotation time per event was reduced from 15.2 s in a traditional manual workflow to 6.3 s using the developed tool, corresponding to a 2.41-fold improvement in labelling efficiency. This translates into an effective throughput of approximately 560 annotated events per hour, compared with 236 events per hour manually. Labelling reliability was assessed through inter-annotator comparison, yielding an agreement level of 95.2%, while expert review showed that the frequency of required corrections decreased by 59% when using the tool. These indicators confirm that the proposed annotation framework significantly improves both the efficiency and accuracy of the dataset creation process.

In total, more than 36 h of recordings were processed and annotated from eight different locations in the Žilina region. The initial dataset contained approximately 150,000 raw detection events, which were subsequently filtered and verified to produce a reduced set of around 50,000 valid vehicle records. Each detection was manually assigned to one of five predefined classes based on video analysis and vehicle characteristics:

- Class 1: 18,396 records—Motorcycles and passenger cars.
- Class 2: 2214 records—Vans, delivery vehicles, and light-duty trucks (up to 3.5 tons).
- Class 3: 1467 records—Standard trucks and buses.
- Class 4: 3112 records—Heavy-duty trucks and articulated lorries.
- Class 0: 24,371 records—Non-vehicle detections, noise, or ambiguous events (excluded from further analysis).

This structured and balanced dataset serves as a valuable resource for evaluating detection algorithms and training classification models tailored to magnetic-signature-based traffic monitoring.

**Figure 7:**
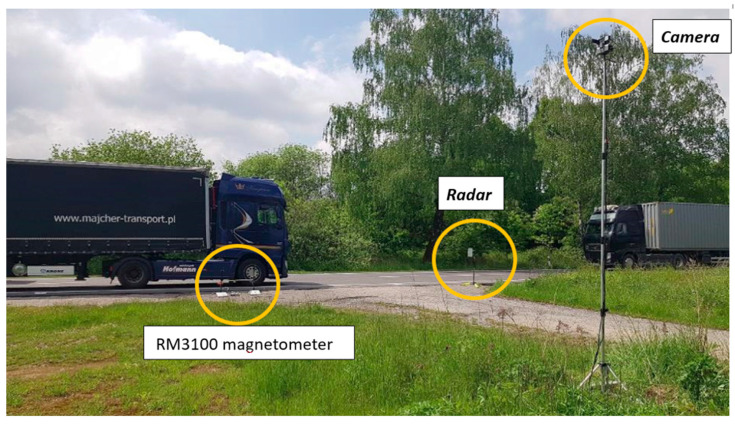
Experimental setup for magnetic traffic monitoring.

To ensure reliable synchronization between different sensing modalities, each measurement site was equipped with a tri-axial magnetometer RM3100, a Sierzega SR4 radar unit, and a high-definition camera mounted on a telescopic mast (Figure 7). The magnetic sensors were embedded at the road surface level close to the lane centreline, while the radar and camera provided complementary reference data used for cross-validation and labelling. This configuration allowed precise temporal alignment of the magnetic signals with radar-based speed and distance information, as well as visual confirmation of vehicle type and trajectory. Such a multimodal setup significantly improved the accuracy of dataset annotation and contributed to the robustness of the generated ground-truth labels.

**Figure 8:**
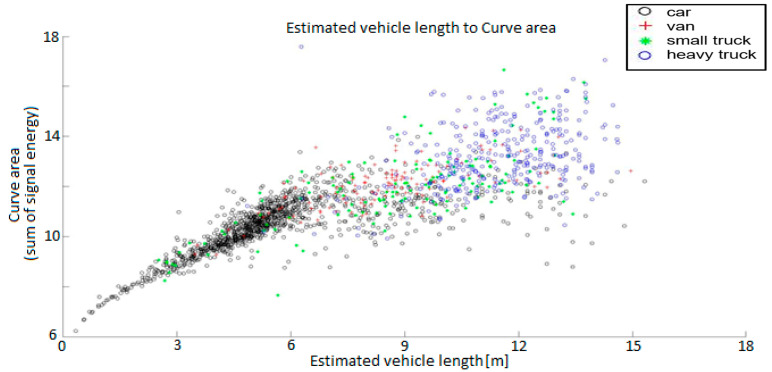
Vehicle category distribution based on the relationship between magnetic signal length and curve area.

**Figure 9:**
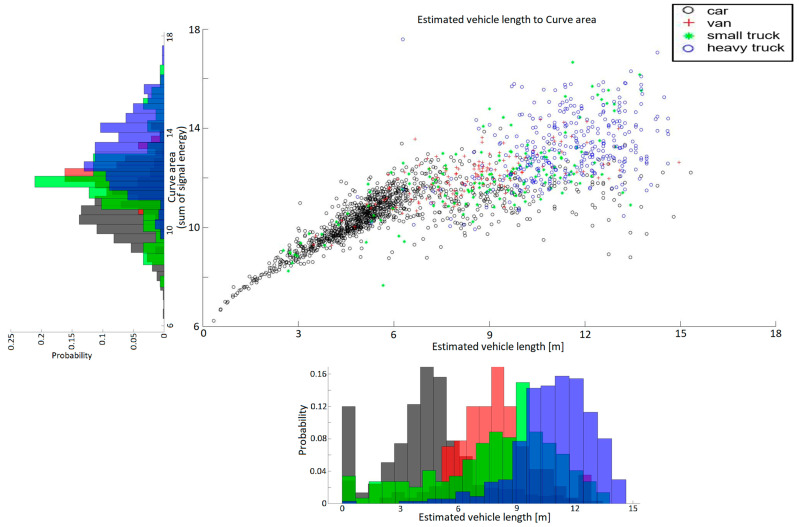
Vehicle category distribution based on the relationship between magnetic signal length and curve area with probability distribution.

Following the completion of data labelling, a statistical evaluation of the annotated dataset was performed to examine the relationships between magnetic signal parameters and the physical characteristics of passing vehicles. This analysis aimed to verify whether the extracted features—such as signal length, transition width, and curve area—carry sufficient discriminative information for reliable vehicle classification. By correlating these parameters across all annotated events, characteristic patterns emerged that reflect the magnetic signature diversity among different vehicle categories. The results, visualized in Figure 8 and Figure 9, provide valuable insight into how specific magnetic features can be used for automated classification and model training within the proposed annotation framework.

To better understand the relationship between the magnetic signal characteristics and the physical properties of detected vehicles, the processed dataset was analyzed using feature correlations derived from the filtered magnetic waveforms. Figure 8 shows the relationship between the estimated vehicle length and the corresponding curve area, representing the integrated magnetic response over the detection interval. A clear correlation can be observed, where larger and heavier vehicles exhibit both longer signal durations and greater magnetic energy. The clustering of points further supports the separability of vehicle categories—passenger cars form a dense cluster in the lower left region, while vans, light trucks, and heavy trucks progressively extend toward higher curve areas and longer magnetic signatures.

Figure 9 complements this analysis by presenting the frequency distribution of vehicle categories with respect to transition width and curve area. The histograms confirm that vehicle classes can be effectively distinguished using these two derived features, forming a suitable basis for subsequent machine learning classification. This statistical visualization demonstrates the discriminative potential of magnetometer-based features and validates the applicability of the developed annotation tool for generating structured datasets used in Intelligent Transportation Systems (ITS) and smart magnetic sensing applications.

The obtained results confirm that magnetic signal parameters—particularly signal length, transition width, and curve area—carry strong discriminative information for identifying vehicle categories in real traffic scenarios. The clustering patterns derived from the RM3100 magnetometer data reveal clear separability between passenger cars, light-duty vehicles, and heavy trucks, which validates the potential of magnetic signatures as a reliable feature source for vehicle classification. By linking each detected event to its synchronized video and radar reference, the developed annotation tool provides a robust framework for generating multimodal datasets with consistent ground truth labels.

These findings are in line with recent research on multimodal annotation and data fusion systems. Similarly to the ezLabel annotation framework for ADAS applications [7], our system demonstrates that the combination of signal-derived and visual features significantly enhances dataset consistency and reduces manual labelling effort. The results also correspond with approaches described in [8], where heterogeneous sensor data—magnetic, radar, and optical—are integrated to improve traffic object classification accuracy. Furthermore, the inclusion of magnetic energy and geometric signal features supports the hybrid sensing concepts discussed in [12], highlighting the role of intelligent preprocessing and feature engineering in machine learning workflows. These parallels indicate that magnetometer-based datasets can effectively complement existing perception systems in modern Intelligent Transportation Systems (ITS), especially when deployed as low-power, cost-effective nodes in smart infrastructure networks.

## 4. Conclusions

This paper introduced an efficient methodology and supporting annotation tool for generating structured datasets from MEMS magnetometer-based sensor systems. The tool facilitates synchronized handling of magnetic, radar, and video data, providing an integrated environment for semi-automatic labelling and validation. Statistical analysis of the annotated dataset confirmed that magnetic waveform parameters such as signal length and curve area exhibit strong correlations with vehicle size and type, making them suitable for feature-based classification and AI model training.

The presented results demonstrate that accurately annotated magnetic datasets are key enablers for the development of smart magnetic sensor applications in ITS and smart city environments. Despite the demonstrated improvements in annotation efficiency and dataset consistency, the proposed system presents several limitations: First, ambiguous or low-quality events still require manual verification, which may introduce subjectivity in isolated cases. Second, the dataset used for evaluation was collected at a limited number of regional sites, and broader geographic validation would be necessary to fully assess generalizability.

The statistical evaluation of the annotated dataset demonstrated that magnetic signal features—such as signal length, transition width, and curve area—exhibit clear separability across vehicle categories, confirming their suitability for feature-based classification. The efficiency study further showed that the proposed annotation tool reduced average labelling time by more than a factor of two and significantly decreased annotation inconsistency, as evidenced by the high inter-annotator agreement and reduced correction rate. These results collectively demonstrate that the developed framework not only streamlines dataset creation but also improves label quality, making it a practical and effective solution for generating multimodal training data in MEMS-based traffic monitoring applications.

In addition to the demonstrated results, it is important to recognize several limitations of the present work. The annotation process still requires manual verification for ambiguous or low-quality events, which introduces occasional subjectivity. Furthermore, the dataset used for evaluation was collected at a limited number of regional sites, which may restrict the generalizability of the findings to broader traffic conditions. Beyond these constraints, the outcomes of this study highlight the wider significance of structured magnetic datasets for emerging research directions in intelligent transportation systems. As multimodal sensing becomes increasingly integrated into roadside infrastructure and autonomous vehicle platforms, the need for reliable, domain-specific annotation tools continues to grow. The proposed framework contributes to this evolution by providing a unified environment for consistent labelling of magnetic, radar, and video data, supporting both traditional signal-processing methods and modern data-driven approaches. By enabling systematic creation of high-quality magnetic signatures linked to verified ground truth, the tool provides a foundation for future research in traffic-flow modeling, sensor-fusion strategies, and the development of robust algorithms for low-power, infrastructure-embedded sensing.

Future work will focus on expanding the tool’s capabilities with adaptive filtering, automated outlier detection, and the integration of machine learning-assisted annotation modules. Such extensions will further improve annotation efficiency and pave the way for scalable, AI-driven traffic monitoring systems based on intelligent magnetic sensing.

## Data Availability

The original contributions presented in this study are included in the article. Further inquiries can be directed to the corresponding author.
